# The Adolescent Microbiome–Gut–Brain Axis: Development, Mechanisms, and Translational Perspectives

**DOI:** 10.3390/nu18172891

**Published:** 2026-09-03

**Authors:** Dolores B. Vazquez-Sanroman, Liam Longaberger, Alondra Chance, Michael Anderson, Nedra Wilson, Gerwald Koehler

**Affiliations:** 1 Department of Anatomy and Cell Biology, Oklahoma State University Center for Health Sciences, 1111 W. 17 Street, Tulsa, OK 74107, USA; 2 Department of Biochemistry and Microbiology, Oklahoma State University Center for Health Sciences, 1111 W. 17 Street, Tulsa, OK 74107, USA

**Keywords:** adolescence, microbiome–gut–brain axis, neurodevelopment

## Abstract

Background: Adolescence represents a pivotal, yet understudied, developmental window in which maturation of the gut microbiome, neural circuits, endocrine systems, and behavioral processes converge. Although early-life microbiota–gut–brain interactions are well established, emerging evidence demonstrates that adolescence constitutes a “second sensitive period” during which microbial composition, metabolic output, and immune function undergo substantial restructuring. These microbial transitions parallel key neurodevelopmental events, including synaptic pruning, dopaminergic refinement, and heightened neuroendocrine activity, suggesting a coordinated microbiome–gut–brain developmental program. Cross-species comparisons reveal broad alignment between rodent and human adolescent milestones, though inconsistencies in development staging present challenges for translational interpretation. Environmental influences—such as diet, stress, antibiotics, and physical activity—further modulate microbial and neural maturation during this period, with potential long-term consequences for cognition, emotion, and stress responsivity. Mechanistically, microbial metabolites, vagal pathways, immune mediators, and steroid hormone interactions constitute key interfaces linking the gut microbiome to adolescent brain function. Recognizing adolescence as a mechanistically rich and plastic phase of gut–brain co-development underscores the need for harmonized methodological approaches and developmental alignment frameworks. Methods-Results: This review synthesizes ontogenetic, mechanistic, and translational evidence to highlight conserved microbiome–gut–brain pathways and identifies opportunities for targeted interventions during this critical developmental stage.

## 1. Introduction

The microbiome–gut–brain axis represents a complex bidirectional network linking the gastrointestinal microbiota to central nervous system (CNS) function through neural, endocrine, immune, and metabolic pathways [1]. Communication along this axis occurs via microbial metabolites and neuroactive compounds that modulate neural signaling, stress physiology, and behavior. In turn, the CNS influences gut motility, secretion, and microbial composition through neuroendocrine and autonomic feedback. This dynamic crosstalk is fundamental for maintaining physiological homeostasis and for guiding neurodevelopmental trajectories across the lifespan. Over the past decade, converging evidence has established the gut microbiota as a key regulator of early-life brain development and long-term mental health [1,2,3]. Microbial colonization begins at birth, coinciding with rapid brain growth and endocrine calibration [4,5]. During this period, microbially derived metabolites such as short-chain fatty acids, tryptophan derivatives, and imidazole propionate enter systemic circulation and influence neuronal differentiation, synaptic organization, and neurotransmitter signaling [6]. Germ-free mouse studies demonstrate that the absence of microbial input leads to widespread alterations in gene expression related to synaptic plasticity, neurotransmission, and stress responsivity, as well as behavioral abnormalities in anxiety and locomotion [7]. These findings establish that microbial signals are indispensable for normal neurodevelopment. While early postnatal development has received the most attention, adolescence is now recognized as an equally critical, yet underexplored, phase of gut–brain interaction [8]. This period is characterized by extensive remodeling of neural circuits, endocrine systems, and social–emotional behavior. Parallel to these neurobiological changes, the gut microbiome undergoes marked compositional and functional transitions that may recalibrate gut–brain signaling [4,9,10]. In rodents, pre-adolescence (postnatal day [P] 21–33) and adolescence (P35–60) [8,11,12] correspond broadly to human late childhood and puberty [8]. Table S1 outlines these developmental stages, highlighting cross-species parallels in microbiome maturation, neural remodeling, vagal activity, and behavioral adaptation. Mechanistically, the gut microbiota influences the adolescent brain through multiple convergent routes. Microbial metabolites function as signaling molecules that modulate neurotransmission and synaptic plasticity, whereas the vagus nerve provides a direct neural conduit that transmits gut-derived signals to brainstem and limbic structures [13]. Disruption of vagal integrity prevents microbiota-driven effects on hippocampal neurogenesis, neuroinflammation, and depressive-like behaviors, underscoring the necessity of gut–brain communication for emotional regulation [14,15,16]. Similarly, germ-free and antibiotic-treated rodents exhibit exaggerated hypothalamic–pituitary–adrenal (HPA) axis responses to stress, demonstrating that microbial input calibrates neuroendocrine stress circuitry [7].

**Figure 1:**
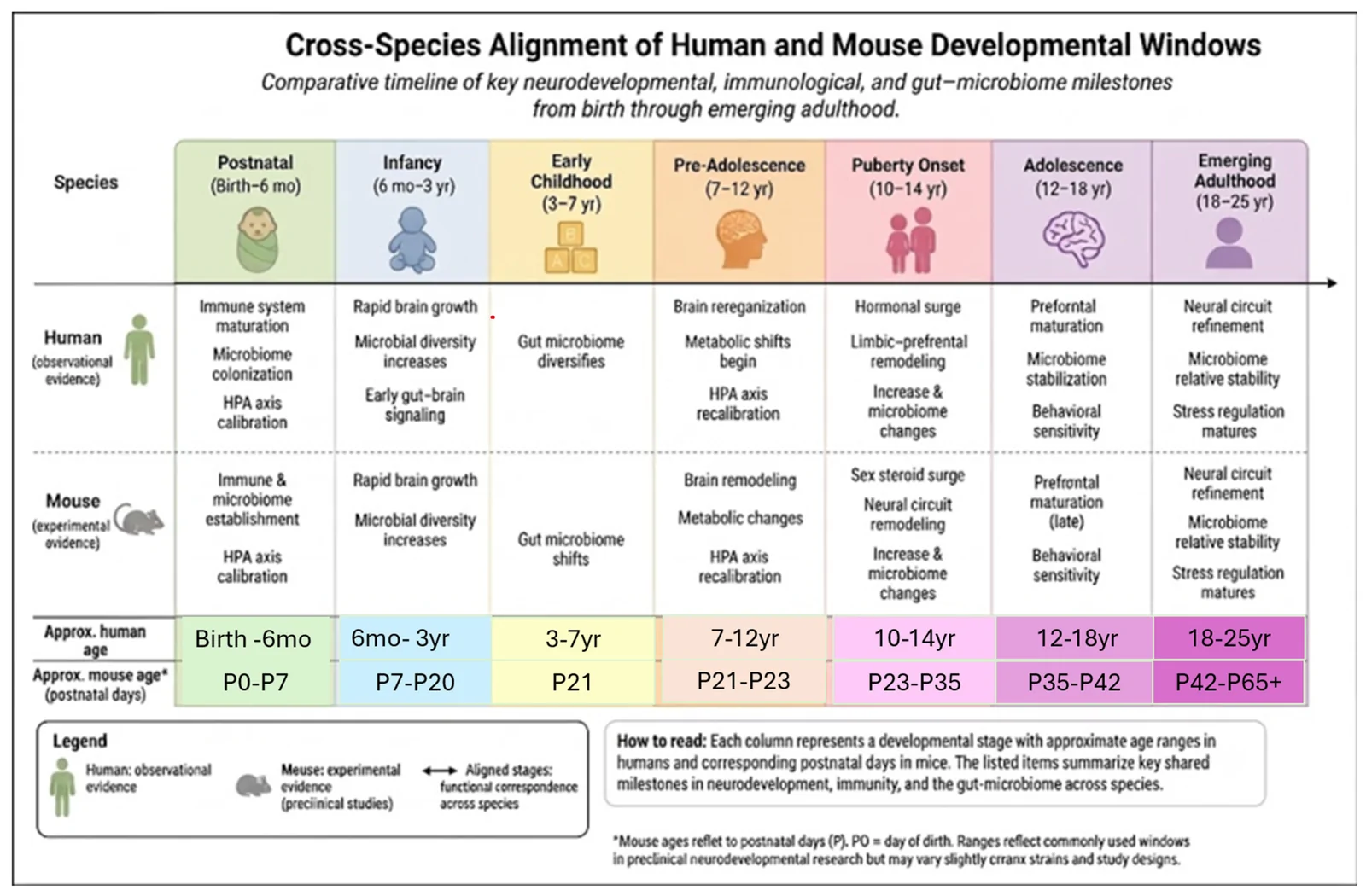
Cross-species alignment of human and mouse developmental windows. Schematic comparison of approximate developmental correspondence between humans and mice across key neurodevelopmental, immune, and gut–microbiome maturation periods. Human developmental milestones represent observational evidence from clinical and population-based studies, whereas mouse milestones represent experimental evidence from preclinical models. The alignment illustrates commonly used translational developmental windows but does not imply identical biological mechanisms or equivalent levels of evidence across species. Mouse ages are expressed as postnatal days (P), where P0 indicates the day of birth (e.g., P0–7 = postnatal days 0–7; P7–20 = postnatal days 7–20). These windows represent approximate developmental periods that may vary depending on strain, sex, and experimental design.

Sex hormones introduce an additional layer of regulation during adolescence. The onset of puberty triggers sharp increases in gonadal steroids that shape both microbiome composition and neural plasticity within stress and social circuits [17,18,19]. Conversely, microbial metabolism contributes to the biotransformation and recycling of steroid hormones, thereby influencing their systemic availability and creating a bidirectional microbiome–endocrine feedback system [20,21]. Nutritional factors, such as omega-3 polyunsaturated fatty acids, further interact with microbial and hormonal signaling to shape sex-dependent trajectories of neuroendocrine development and emotional regulation [7,22]. Collectively, these processes underscore adolescence as a critical developmental window for maturation of the gut–brain axis. This review positions adolescence as a distinct and mechanistically rich phase of gut–brain interaction [10], rather than a simple transition between early life and adulthood [8,12,23]. It integrates developmental and mechanistic frameworks (see Figure 1) to identify conserved microbiome–brain pathways across species. The overarching goal is to delineate how gut microbiome maturation and neural signaling co-develop during adolescence, highlighting both conserved and species-specific mechanisms that influence brain function, stress regulation, and behavior.

## 2. Core Neuroimmune, Endocrine, and Metabolic Pathways of the Microbiome–Gut–Brain Axis

### 2.1. Foundational Mechanisms of the Microbiome–Gut–Brain Axis

The microbiome–gut–brain axis refers to a bidirectional communication network in which gut microorganisms and the metabolites they produce interact dynamically with the enteric and central nervous systems, as well as with immune and endocrine pathways. In this system, gut microbes generate a wide range of bioactive compounds that can influence neural signaling and physiological processes within the gastrointestinal tract and the brain. These microbial signals are transmitted through the enteric nervous system and integrated with central nervous system activity, while also engaging immune responses and hormonal (endocrine) signaling pathways. Together, these interconnected components continuously regulate and influence one another, forming a complex system through which gut microbiota can impact brain function and, conversely, the brain can shape gut microbial composition and activity [24,25,26,27]. This system integrates neural signaling via vagal and spinal afferents, immune pathways involving cytokines and microglial activation, endocrine communication mediated by the HPA axis and enteroendocrine hormones, and a wide array of microbially derived metabolites that reach the brain through the circulation. Together, these communication routes enable gut microbial communities to shape neurodevelopment, sensory processing, stress responsivity, and emotional and cognitive function across the lifespan [19].

### 2.2. Neural Communication Pathways

The enteric nervous system (ENS) serves as the primary interface linking luminal microbial activity with the central nervous system (CNS). Microbial signals modulate vagal afferent excitability, influencing limbic and brainstem circuits that regulate anxiety, arousal, and autonomic state [28]. Experimental studies show that gut microbes regulate ENS development, synaptic signaling, and motility via microbe-derived metabolites and pattern-recognition receptor activation [29,30]. Vagal pathways are also required for several microbiome-mediated behavioral effects, including anxiolytic responses to probiotic treatment [31,32].

Critically, these neural pathways are not static but undergo a profound functional reorganization during adolescence. Vagal tone, the functional readout of parasympathetic efferent activity and a key indicator of gut–brain circuit integrity, is uniquely plastic during this transitional phase [2,33,34,35]. Emerging translational frameworks indicate that the adolescent nervous system exhibits heightened vulnerability to environmental and microbial disruptions; transient exposures to antibiotics [36], dietary shifts, or severe stressors during this sensitive window can arrest the normal development of autonomic networks and lead to long-term reductions in vagal integrity [2]. This developmental tuning of vagal circuits serves as an important translational pathway, as diminished vagal tone is a common feature in adolescent-onset neuropsychiatric disorders [37].

### 2.3. Immune and Inflammatory Signaling

Immune communication represents a major route through which the microbiome influences brain development. Microbial products, such as lipopolysaccharide, peptidoglycan, and flagellin, activate host immune receptors, thereby shaping cytokine profiles that alter microglial maturation, synaptic pruning, and neuroinflammatory tone [38,39]. Germ-free mice exhibit pronounced microglial deficits, including altered morphology and impaired immune responsiveness, that are rescued by short-chain fatty acid supplementation, demonstrating the essential role of microbial metabolites in immune-brain signaling [40]. From a developmental perspective, adolescence is increasingly recognized as a period of profound neuroimmune remodeling, a phenomenon termed “immunoadolescence” [41,42]. During this stage, both central and peripheral immune systems undergo [42] distinct maturation processes that directly affect synaptic pruning, myelination, and the refinement of frontolimbic circuitry [23,42,43,44]. Rather than being fixed in infancy, the microglial landscape continues to be sculpted by microbial inputs well into the adolescent period [22]. Consequently, adolescent gut dysbiosis can trigger low-grade systemic inflammation and alter central cytokine signaling [38,39,45], thereby derailing standard neuroimmune trajectories and increasing the host’s long-term vulnerability to neuroinflammatory conditions and affective disorders [2,27,45].

### 2.4. Endocrine and HPA-Axis Communication

Microbiota–HPA interactions are established early in life and continue to influence stress responsivity across development. Germ-free animals exhibit exaggerated corticosterone responses to stress, which can be normalized by early-life microbial reconstitution [46]. Gut microbes also regulate secretion of enteroendocrine hormones such as GLP-1, PYY, and ghrelin, which modulate appetite, reward circuitry, and metabolic homeostasis [28,32,47]. Through these endocrine routes, microbial communities shape both basal neuroendocrine function and plasticity of the developing stress axis [19].

This neuroendocrine calibration is highly specific to the adolescent stage, which represents a critical window of vulnerability for frontolimbic networks [2,18,48]. The HPA axis undergoes significant structural and functional maturation during puberty, marked by changes in glucocorticoid receptor expression and an elevated sensitivity to stressors [49]. Dysregulation or composition shifts in the gut microbiota during this period can lead to chronic HPA-axis hyperactivation and elevated circulating glucocorticoids, which preferentially damage vulnerable, developing front limbic structures involved in emotional processing and mood regulation [50,51]. This interface provides a crucial translational mechanism explaining the sharp, adolescent-specific rise in stress-induced disorders like major depression.

### 2.5. Metabolic and Bioactive Molecule Signaling

Microbial metabolites constitute a diverse chemical interface between the gut lumen and the brain. The SCFAs including acetate, propionate, and butyrate cross the blood–brain barrier and regulate gene expression, neurotransmitter synthesis, microglial activation, and neuroplasticity [52,53,54] Tryptophan-derived indoles influence serotonergic signaling and aryl hydrocarbon receptor pathways, impacting mood and stress regulation [55]. Secondary bile acids, phenolic metabolites, and trimethylamine-N-oxide (TMAO) further contribute to neuroimmune and metabolic communication [26,55,56]. Together, these bioactive compounds form a metabolite-based “chemical connectome” linking gut microbial activity to CNS function [55,57]. Furthermore, the microbiome derived metabolites undergo significant restructuring during adolescence. As the gut microbiome transitions toward a stable, adult-like configuration, the baseline production and relative ratios of SCFAs, tryptophan derivatives, and secondary bile acids fluctuate drastically in response to pubertal shifts [54,58]. These dynamic shifts in the metabolic milieu occur precisely when blood–brain barrier permeability and central receptor profiles are being recalibrated [11,23,38]. Disruptions to these specific metabolite pools during the adolescent window can alter local signaling within midbrain reward structures and cortical domains, laying down enduring behavioral and physiological signatures that persist into adulthood [59].

### 2.6. Relevance for Neurodevelopment and Existing Gaps

Across early life, these pathways collectively shape neural circuit formation, stress-axis calibration, emotional behavior, and cognitive trajectories, as shown by germ-free animal studies, antibiotic depletion models, and metabolite-supplementation experiments. However, the majority of mechanistic research centers on infancy and early childhood. Far less is known about how these communication routes function during adolescence, a second sensitive developmental and microbial restructuring period characterized by heightened neuroplasticity, pubertal endocrine transitions, and major shifts in gut microbial composition [8]. Understanding how neural, immune [27], endocrine, and metabolic signaling pathways operate during this adolescent period is therefore critical for elucidating how environmental exposures and microbiome-targeted interventions [60] influence long-term neurobiological and mental health outcomes. Together, these foundational neural, immune, endocrine, and metabolic pathways demonstrate how gut microbial communities shape brain development and function across the lifespan.

The following section focuses on these adolescent-specific mechanisms, examining how vagal signaling, immune modulation, hormonal dynamics, and microbially derived metabolites interface with the maturing brain during this second sensitive period of microbiome–gut–brain plasticity.

### 2.7. Experimental Approaches Used to Investigate the Adolescent Microbiome–Gut–Brain Axis

Investigating the microbiome–gut–brain axis during adolescence requires the integration of complementary experimental approaches capable of resolving microbial composition, functional outputs, host physiological responses, and behavioral consequences. Because adolescence represents a developmental period characterized by simultaneous maturation of neural circuits, endocrine systems, immune function, and microbial communities, studies have increasingly adopted multidisciplinary approaches combining animal models, human cohort studies, microbiome profiling, metabolomics, and neurobehavioral assessments.

#### 2.7.1. Preclinical Approaches for Establishing Causality

Animal models remain essential for determining causal relationships between microbial alterations and neurodevelopmental outcomes because they allow controlled manipulation of microbial communities, environmental exposures, and developmental timing. Germ-free (GF) animals, which are raised in sterile conditions without microbial colonization, represent one of the most extensively utilized models to investigate microbiome-dependent regulation of brain development [61]. Early studies demonstrated that absence of commensal microbiota alters hypothalamic–pituitary–adrenal (HPA) axis maturation, increases stress responsivity, modifies hippocampal expression of neurotrophic factors, and influences anxiety-related behaviors. Importantly, microbial colonization during specific developmental windows can partially reverse these abnormalities, demonstrating that microbial signals contribute to developmental programming of stress and emotional circuits [46,62]. Antibiotic-induced microbiome depletion provides an additional experimental strategy to evaluate how transient or prolonged disruption of microbial communities influences brain function [39]. Unlike germ-free models, antibiotic approaches allow investigation of microbiome perturbations after normal colonization has occurred, more closely resembling environmental exposures experienced by humans [36,63] Antibiotic-treated animals have demonstrated alterations in neurogenesis, microglial activation, neurotransmitter metabolism, and reward-related behaviors, supporting the concept that microbiome disruption during sensitive developmental periods may influence neural plasticity [15,39].

Fecal microbiota transplantation (FMT) and microbial recolonization approaches provide additional evidence for causal relationships by determining whether behavioral or physiological phenotypes can be transferred through microbial communities [64]. FMT studies have demonstrated that microbial composition from stressed or resilient donors can influence recipient behavior, hippocampal neurogenesis, inflammatory pathways, and stress responses, suggesting that microbial communities contain functional information capable of modulating brain-related phenotypes [65].

Dietary manipulation, including high-fat diet exposure, fiber supplementation, omega-3 fatty acid interventions, and targeted prebiotic or probiotic administration, represents another major experimental strategy. These approaches allow researchers to determine how environmental factors alter microbial ecology and whether restoration of beneficial microbial metabolites can improve neurobehavioral outcomes. Dietary interventions [60] influence the production of SCFAs, bile acid metabolites, tryptophan derivatives, and immune mediators that regulate neuronal signaling and neuroplasticity [66].

#### 2.7.2. Behavioral, Molecular, and Physiological Assessments

Experimental studies investigating microbiome–brain interactions commonly combine microbial manipulation with behavioral phenotyping and molecular analyses. Behavioral paradigms frequently include assays of anxiety-like behavior (elevated plus maze, open field), depressive-like behavior (forced swim or sucrose preference), cognition and memory (novel object recognition, Morris water maze), social behavior, and reward-related responses. These behavioral measures are integrated with molecular assessments examining inflammatory cytokines, microglial activation, neurotrophic factors such as brain-derived neurotrophic factor (BDNF), neurotransmitter systems, synaptic proteins, and HPA-axis hormones [6,65,67,68]. During adolescence, these approaches are particularly important because reward circuits, including dopaminergic pathways within the ventral tegmental area, nucleus accumbens, and prefrontal cortex, undergo extensive remodeling. Microbiome manipulation studies targeting these circuits have demonstrated changes in dopamine signaling, reward sensitivity, and susceptibility to addictive behaviors, highlighting the importance of developmental timing when interpreting microbiome–brain interactions [69,70].

#### 2.7.3. Human Approaches and Translational Methodologies

Human research of the adolescent microbiome–gut–brain axis rely primarily on observational cohorts, longitudinal developmental studies, and integrative multi-omics approaches [71]. Fecal microbiome profiling using 16S rRNA sequencing or shotgun metagenomic sequencing represents the most common methodology for characterizing microbial composition and diversity. These approaches are frequently combined with dietary assessments, anthropometric measurements, endocrine profiling, inflammatory markers, cognitive testing, and behavioral questionnaires to examine associations between microbial features and neurodevelopmental outcomes. Longitudinal human studies are particularly valuable during adolescence because microbial communities, hormone levels, immune responses, and brain maturation change rapidly during this period. Studies examining developmental transitions have demonstrated that puberty is associated with alterations in microbial diversity [43,71], taxonomic composition [8], and microbial metabolic capacity, suggesting that endocrine maturation represents an important factor shaping microbiome trajectories [58].

More recently, multi-omics strategies integrating metagenomics, metabolomics, transcriptomics, proteomics, and neuroimaging have emerged as powerful approaches for identifying mechanistic links between microbial activity and brain function. These approaches move beyond taxonomic descriptions by examining microbial functional capacity and host biological responses. For example, metabolomic analyses have identified microbial-derived compounds, including SCFAs [7,40,52,53], indole derivatives, and secondary bile acids [54,58], as potential mediators connecting microbial communities with immune signaling [54], endocrine regulation [7], and neuronal function [34,72].

Despite significant advances, methodological variability remains a major challenge in the field. Differences in sequencing platforms, bioinformatic pipelines, microbial taxonomy databases, dietary assessment methods, developmental staging criteria, and behavioral measurements complicate direct comparison across studies. Furthermore, human studies are often limited by confounding variables including diet, medication exposure, socioeconomic factors, physical activity [73], and environmental influences. Therefore, standardized longitudinal frameworks incorporating microbiome composition, microbial function, host metabolism, endocrine status, immune markers, and neurobehavioral outcomes will be essential for advancing translational understanding of adolescent microbiome–gut–brain interactions.

## 3. Adolescence as a Second Critical Window of Gut–Microbiome–Brain Axis Development

Adolescence is increasingly recognized as a second sensitive period in the development of the gut–microbiome–brain axis, following early life and marked by renewed biological plasticity. This stage is characterized by tightly coordinated maturation across neural, endocrine, immune, and microbial systems, occurring alongside synaptic refinement, pubertal hormonal transitions, and substantial restructuring of the gut microbiome [48]. When considered within a developmental framework that spans early-life ontogeny through adolescence, these parallel transitions reveal continuity in timing-sensitive microbiome–brain communication rather than isolated critical windows. Cross-species evidence further demonstrates conserved alignment of microbial, neuroendocrine, and neural developmental milestones, strengthening the translational relevance of adolescent animal models for human health. Together, these findings position adolescence as a biologically privileged window during which environmental exposures can exert lasting effects on gut–brain signaling, providing the foundation for the following section, which delineates the mechanistic neural, immune, endocrine, and metabolic pathways through which microbial activity shapes the maturing brain.

### 3.1. Pubertal Maturation and the Adolescent Microbiome–Gut–Brain Axis

While early-life stress and maternal psychological adversity establish a foundational trajectory for infant microbial composition and inflammatory tone [74], adolescence represents a distinct, second major developmental window characterized by substantial restructuring of both the gut microbiome and brain circuitry [23,75]. This renewed period of plasticity in microbiome–brain communication is highly sensitive to environmental inputs like chronic stress, dietary imbalance, or antibiotic exposure, which can induce long-term alterations in neural circuit function and behavior [8,76].

This sensitive period operates through several tightly coordinated, pubertal-specific processes:

#### 3.1.1. Shifts in Microbial Diversity & Sex Steroids

Driven by the systemic surge of gonadal hormones, the adolescent gut microbiota undergoes a secondary successional shift, transitioning toward an adult-like state with expanded metabolic capacity [21,75]. Sex steroids such as testosterone, progesterone, and estradiol contribute directly to the divergence of sex-specific microbial profiles [21,77], while modifying the ratio of core phyla (*Bacteroidetes* and *Firmicutes*) alongside structural changes in the intestinal mucosa. Conversely, gut microbes actively modulate steroid hormone metabolism through enzymatic modification, enterohepatic circulation, and microbial biotransformation of host hormones [77].

#### 3.1.2. Metabolite Production Dynamism

As the microbial community reorganizes, its functional chemical output shifts in tandem. The production of microbially derived neuroactive molecules most notably SCFAs; acetate, propionate, butyrate) and tryptophan metabolites (indoles, serotonin precursors) fluctuates dramatically. Because the adolescent blood–brain barrier is undergoing regulatory maturation, these shifting metabolite pools exert a disproportionately high influence on central neurotransmitter synthesis, epigenetic programming, and myelin sheath maintenance [38].

#### 3.1.3. Adolescent Vagal Signaling

Autonomic communication via the vagus nerve undergoes extensive physiological tuning during puberty [35]. Vagal afferent sensitivity is highly plastic during this second window; microbial shifts can alter the expression of vagal mechanoreceptors and chemoreceptors in the enteric nervous system [78], altering baseline vagal tone and disrupting top-down cognitive control and emotional reactivity [8,32,35].

#### 3.1.4. Neuroimmune Interactions & Pruning

Unlike early-life neurodevelopment, adolescent neuroimmune interactions are dominated by the active refinement and pruning of synaptic networks, dopaminergic system refinement, and white matter maturation [79]. During this period of “immunoadolescence,” resident microglia remain highly sensitive to gut-derived signals [42]. Pubertal gut dysbiosis can trigger peripheral immune activation and systemic cytokine release [1], disrupting microglial-mediated synaptic pruning in frontolimbic circuits [33,79] and derailing healthy emotional and cognitive maturation [2,42].

#### 3.1.5. HPA Axis Reactivity

The hypothalamic-pituitary-adrenal (HPA) axis experiences a dramatic calibration during puberty, resulting in heightened baseline stress responsivity and prolonged recovery times relative to both children and adults. The adolescent gut microbiota plays a vital role in setting the threshold for this reactivity; pubertal dysbiosis or microbial distress amplifies this natural neuroendocrine hyper-responsiveness, leading to sustained glucocorticoid exposure that targets vulnerable, developing brain regions like the hippocampus and amygdala [80].

Collectively, these findings position the microbiome–gut–brain axis as a developmentally dynamic system characterized by two principal sensitive periods: early life, when microbial colonization shapes foundational neuroimmune architecture, and adolescence, when hormonal maturation and environmental exposures converge with microbial restructuring to refine neural and behavioral outcomes. These temporally distinct but mechanistically overlapping phases provide a framework for comparative developmental analysis across species and form the basis for examining cross-species alignment in microbiome–brain co-development in the following section.

### 3.2. Cross-Species Alignment of Microbial and Neural Developmental Windows

Understanding how developmental periods align across species is essential for interpreting microbiome–gut–brain research and improving translational validity. Rodents remain indispensable for mechanistic studies, but their rapid neurodevelopmental tempo, condensed lifespan, and accelerated microbial maturation preclude straightforward chronological comparisons with humans. Translation, therefore, requires aligning developmental windows using physiological, neuroendocrine, and microbial signatures, an approach strongly emphasized in recent comprehensive review [11,81].

Rodent pre-adolescence begins at weaning (P21) (see Figure 1), marking a foundational biological transition involving autonomy from maternal nutrition, rapid hippocampal plasticity, and significant restructuring of the gut microbiome [30]. Microbiota-derived metabolites, such as indoxyl sulfate and trimethylamine-N-oxide, are detectable in the forebrain by P21, indicating early biochemical gut–brain communication [82]. Recent work identifies narrower sensitivity windows within this stage. Lynch et al. demonstrated that antibiotic exposure during P21–27 yields long-lasting effects on microglial morphology and myelin-related gene expression [3], effects far more pronounced than disruptions at earlier or later developmental windows. Complementary enteric nervous system (ENS)-focused findings show that germ-free mice colonized at weaning, but not adulthood, regain normal gastrointestinal motility and ENS gene expression [30,63], demonstrating parallel CNS–ENS dependence on timely microbial signaling.

Rodent adolescence spans roughly P21–65, but functional adolescence, anchored by endocrine transitions, heightened sociability, risk-taking, and limbic–prefrontal remodeling, is concentrated between P35 and P60 [16,83]. Pubertal onset in males occurs around P42 ± 3 [84]. Longitudinal disease models offer further temporal insight. Prenatally androgenized mice (Polycystic Ovarian Syndrome-like phenotype) exhibit microbiome alterations from juvenile stages through adulthood showing evidence of mechanistic endocrine–microbial tracking across development (see Figure 1).

Across the lifespan, microbial richness increases and stabilizes around adolescence, accompanied by functional shifts in carbohydrate, fiber, and vitamin metabolism [85]. Emerging adulthood remains microbiologically plastic. Mohr et al. found substantial shifts in community composition, Prevotella/Bacteroides (P/B) ratios, and metabolomic markers within a cohort of college students [86]. Collectively, convergent neurobiological, endocrine, immunological, and microbiome-developmental evidence supports aligning the early post-weaning/juvenile period (approximately P21–33) with human late childhood (7–12 years) and the mid-to-late rodent adolescent period (approximately P35–60) with human adolescence (12–18 years). These derived windows are consistent with contemporary lifespan frameworks emphasizing timing-sensitive microbiome–brain–immune interactions [1,8,11,16,86,87]. Because rodents and humans progress through partially overlapping but distinct developmental timelines, Figure 1 provides a visual alignment of key maturational windows, including weaning, pre-adolescence, pubertal onset, and functional adolescence. to anchor the translational framework for this section.

### 3.3. Environmental Modulators and Interventions in Adolescent Plasticity: Preclinical vs. Clinical Evidence

Adolescence represents a sensitive neurodevelopmental window marked by extensive remodeling of prefrontal, limbic, reward, and stress-regulatory circuits [44]. In parallel, neuroimmune and microbiome systems exhibit heightened plasticity, with increased microglial remodeling, shifts in microbial composition, and marked pubertal restructuring [22,33,88]. These convergent developmental processes make the adolescent gut–brain axis especially sensitive to environmental inputs, as microbial metabolites reach the maturing brain in age-dependent patterns [82].

#### 3.3.1. Preclinical Evidence (Animal Models)

**Figure 2:**
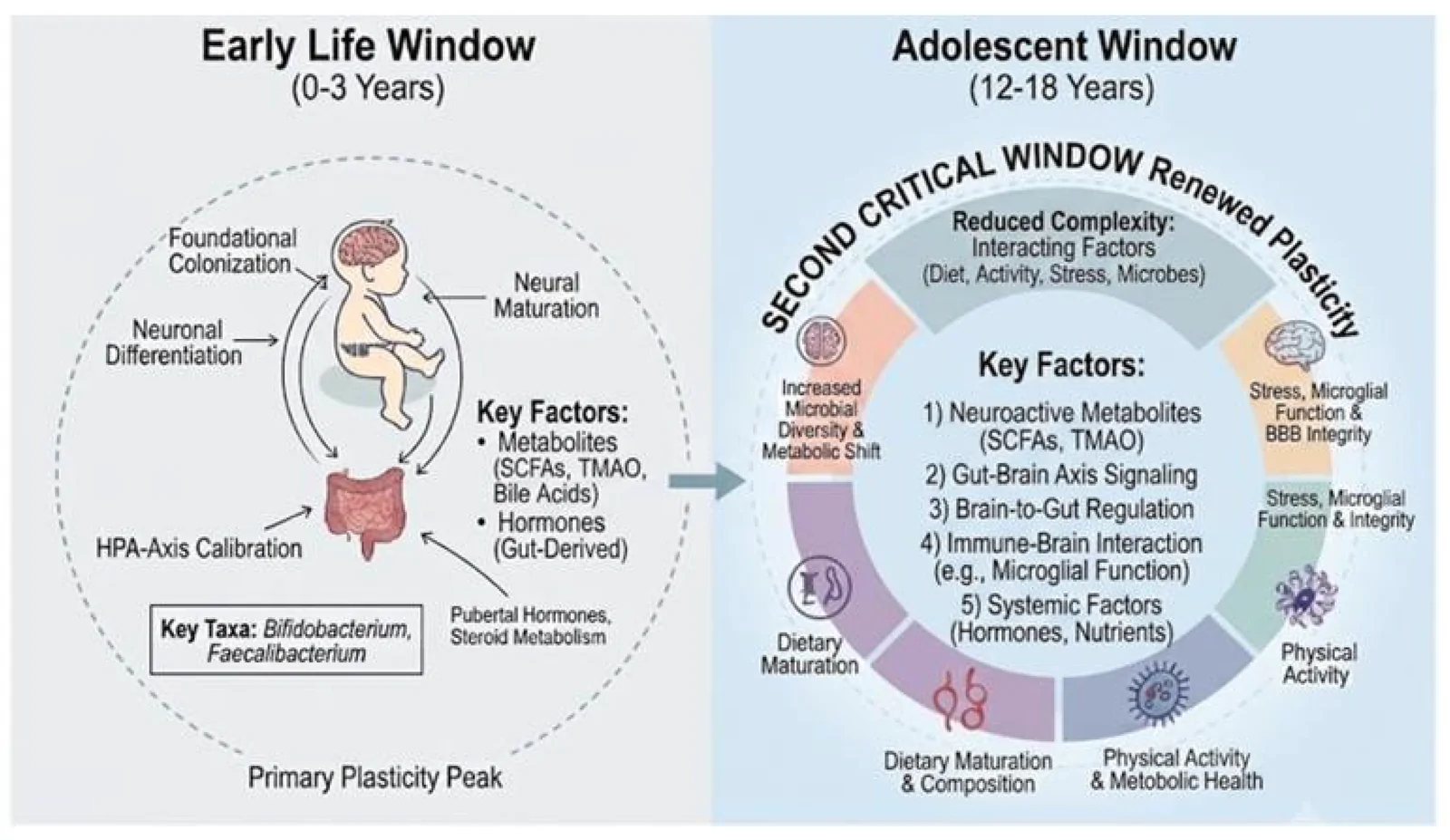
Critical windows of gut–brain axis plasticity across human development. Schematic representation depicting the two primary developmental windows of gut–brain axis maturation and plasticity. Early Life Window (0–3 Years, Left Panel): During this Primary Plasticity Peak, reciprocal bidirectional signaling (curved arrows) occurs between the central nervous system and the gastrointestinal tract, driving foundational microbial colonization, neuronal differentiation, neural maturation, and hypothalamic-pituitary-adrenal (HPA)-axis calibration. These processes are modulated by key factors including gut-derived hormones, pubertal/steroid metabolism, and neuroactive metabolites (e.g., SCFAs, trimethylamine N-oxide [TMAO], and bile acids), with early microbial composition predominantly enriched in key taxa such as *Bifidobacterium and Faecalibacterium*. Adolescent Window (12–18 Years, Right Panel): A large central arrow indicates the temporal transition into a Second Critical Window characterized by Renewed Plasticity. During adolescence, interacting environmental and physiological inputs—represented in the outer segmented wheel—including dietary maturation and composition, physical activity and metabolic health, stress responses, microglial function, and blood–brain barrier (BBB) integrity, converge to refine gut–brain network stability. The inner hub outlines the five core operational mechanisms mediating this renewed plasticity: (1) neuroactive metabolites (SCFAs, TMAO), (2) gut-to-brain signaling, (3) brain-to–gut regulation, (4) immune–brain interactions (e.g., microglial function), and (5) systemic factors (hormones, nutrients).

Mechanistic insights from rodent models demonstrate that environmental challenges including dietary shifts, stress exposure, and antibiotic use fundamentally alter neurodevelopment through distinct microbiome–gut–brain pathways. Preclinical studies show that high-fat or nutrient-poor diets reduce microbial diversity and promote inflammatory signaling that disrupts rodent cognition and emotional behavior [89]. Specifically, experimental omega-3 fatty acid deficiency in adolescent mice induces long-lasting shifts in microbial composition, impairs social behaviors, and heightens stress responsivity, whereas dietary supplementation increases beneficial taxa like *Bifidobacterium* and *Lactobacillus* (See Figure 2) [22]. Controlled psychosocial stress paradigms in rodents similarly cause structural disruptions, including increased intestinal permeability (“leaky gut”) and long-term hyperreactivity of the HPA axis [51,76]. Conversely, exercise regimens in rodents increase short-chain fatty acid (SCFA)-producing taxa (e.g., *Lactobacillus*, *Bifidobacterium*, and *Firmicutes*) [90], strengthen the gut barrier, and activate neurotrophic pathways that rescue hippocampal plasticity [41,91].

At a molecular level, animal models show that these environmental disruptions converge on common mechanisms: reduced SCFA production, altered indole and phenolic profiles, impaired microglial maturation, and dysregulation of HPA-axis development [88] (see Figure 2). Notably, microbially derived metabolites, including trimethylamine *N*-oxide (TMAO) [56], phenylacetyl glycine, imidazole propionate, and 3-indoxyl sulfate, have been detected directly within the developing rodent brain, exhibiting age-dependent variations that suggest a direct role in neural circuit refinement [82].

To buffer these disruptions, preclinical microbiome-targeted interventions [60] show great promise. Administration of specific psychobiotic strains, such as *Bifidobacterium longum* and *Lactobacillus rhamnosus*, successfully modulates rodent HPA-axis activity, neuroinflammation, and neurotransmitter signaling, thereby reducing anxiety- and depression-like behaviors [24,92]. Furthermore, symbiotic combinations (e.g., *B. longum* + galacto-oligosaccharides; *L. rhamnosus* + inulin; *B. bifidum* + fructo-oligosaccharides) enhance these protective effects. Mechanistic testing reveals that these symbiotic significantly boost the production of bioactive metabolites like butyrate, acetate, indoles, and secondary bile acids; these molecules strengthen intestinal tight junction assembly while dampening microglial activation and attenuating central inflammatory responses in the brain [29,55,88].

#### 3.3.2. Clinical and Observational Evidence (Human Studies)

In human adolescents, observational cohorts and clinical trials mirror these upstream preclinical patterns, shifting the focus toward systemic biomarkers, structural correlations, and therapeutic interventions. Human epidemiological data consistently links poor dietary patterns during adolescence to systemic inflammation and reduced gut diversity, which correlates with altered cognitive performance and higher rates of mood dysregulation [89]. Longitudinal studies of adolescents undergoing psychosocial stress exhibit distinct shifts in microbial taxonomic profiles, coupled with elevated peripheral cytokines and altered cortisol awakening responses, demonstrating a clear disruption to HPA axis development and function (see Figure 2) [93]. Furthermore, clinical trials assessing physical activity in youth confirm that exercise increases health-associated, SCFA-producing microbes in the human gut [73,84]. This functional expansion of the microbiome correlates strongly with improved executive function, enhanced anti-inflammatory systemic signaling, and elevated circulating brain-derived neurotrophic factor (BDNF) levels [91].

Translating these findings into clinical practice, microbiome-targeted interventions show immense promise for buffering adolescent stress responsivity and supporting emotional and cognitive development [60]. Clinical evaluations of probiotic, psychobiotic, and synbiotic formulas in youth populations demonstrate an ability to manage anxiety and depressive symptoms by targeting the same underlying systemic networks found in animal studies—namely, reducing peripheral neuroinflammation, stabilizing HPA axis reactivity, and optimizing systemic metabolic and tryptophan profiles [51,76]. By utilizing targeted prebiotics and probiotics to augment bioactive metabolites like SCFAs and indole derivatives, these translational therapies help restore human intestinal epithelial barrier integrity, reduce systemic permeability, and downregulate systemic inflammatory cascades that would otherwise compromise adolescent neurodevelopment [52,84,94,95].

As adolescence marks a period of renewed plasticity in the gut–microbiome–brain axis, it becomes essential to understand the mechanistic routes through which these microbial signals influence developing neural and endocrine systems. Communication between the microbiome and the brain occurs through multiple intertwined pathways including vagal and enteric circuitry, immune and inflammatory cascades, and endocrine and metabolic signaling—that collectively shape neurodevelopmental trajectories. Microbially derived metabolites such as short-chain fatty acids, indoles, bile acids, amino-acid derivatives, and steroid-modulating enzymes exert potent effects on microglial maturation, synaptic remodeling, stress-axis calibration, and sex-hormone dynamics. During adolescence, when limbic–prefrontal networks and HPA-axis responsivity are undergoing active refinement, these microbial signals may have disproportionately strong and lasting effects on cognitive, emotional, and metabolic outcomes. The following section delineates these mechanistic interfaces, emphasizing how neural, endocrine, and immune pathways translate microbial activity into brain-relevant signals during this critical developmental window (see Figure 2).

## 4. Molecular and Neural Pathways That Mediate Gut–Brain Communication During Adolescent Development

**Figure 3:**
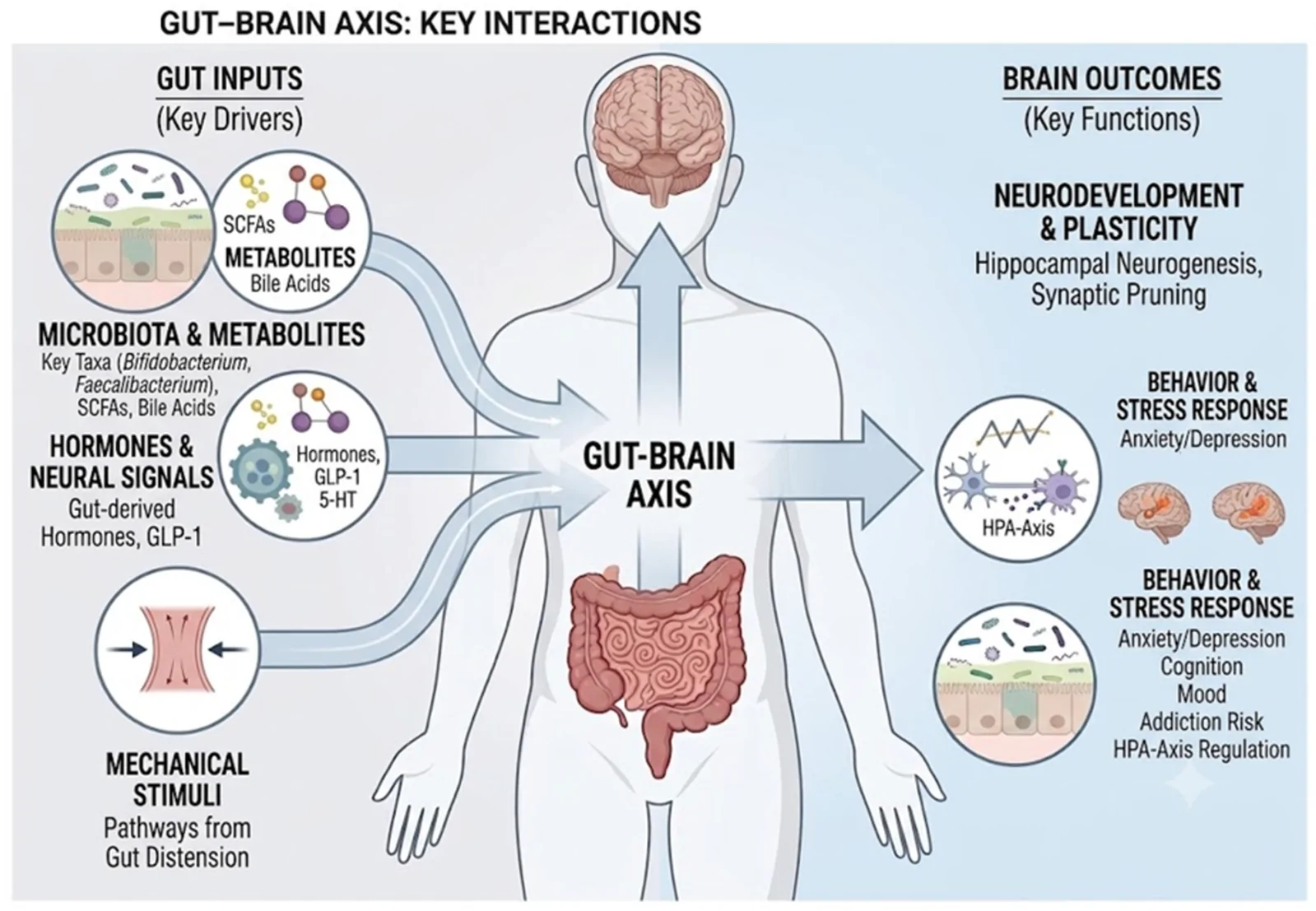
Mechanistic framework of gut inputs and adolescent brain outcomes. Schematic model illustrating the major gut-derived pathways influencing central nervous system trajectories via the gut–brain axis. Gastrointestinal inputs (left) are categorized into three core drivers: (1) microbiota and metabolites, where key taxa (e.g., *Bifidobacterium* and *Faecalibacterium*) generate functional neuroactive molecules such as SCFAs and bile acids; (2) hormones and neural signals, driven by enteroendocrine secretion of regulatory peptides and neurotransmitters including GLP-1 and serotonin (5-HT); and (3) mechanical stimuli, mediated by visceral mechanosensory response pathways activated by gut distension. These distinct peripheral signals converge at the central gut–brain axis interface (center), driving bidirectional communication that directly influences adolescent neurodevelopment and function (right). Key downstream outcomes include structural and functional neurodevelopment and plasticity (e.g., hippocampal neurogenesis and synaptic pruning) as well as modulation of long-term behavior and stress responsiveness via HPA-axis regulation, shaping vulnerability to anxiety, depression, mood alterations, and addiction risk.

The Vagus nerve (VN) functions as the principal sensory axis by which the gastrointestinal (GI) tract communicates with the central nervous system (CNS), continuously conveying mechanical, chemical, and microbial-derived information [32]. The afferent fibers that comprising approximately 80% of all vagal axons originate in the nodose ganglion, projecting to the caudal Nucleus tractus solitarius (NTS). There, mechanosensitive endings transduce gastric distension and intestinal stretch into glutamatergic signals, thereby communicating visceral state to the brainstem. Chemosensory signals in the gut are primarily detected and transmitted by specialized epithelial cells, particularly enteroendocrine (EEC) cells and enterochromaffin (EC) cells, which function as key interface elements between the gut lumen and host signaling systems. These cells express a range of nutrient-, metabolite-, and microbial-sensing receptors that allow them to detect chemical changes in the intestinal environment, including dietary components, microbial-derived metabolites, and microbial-associated molecular patterns. Upon stimulation, EEC and EC cells release a variety of signaling molecules such as hormones (e.g., peptide YY and glucagon-like peptide-1) and neurotransmitter-like mediators such as serotonin, which can act locally on enteric neurons or signal systemically through endocrine and vagal pathways. Through these mechanisms, EEC and EC cells translate luminal chemical information into neural, immune, and endocrine signals that contribute to gut–brain communication and overall physiological regulation [32,66,96]. Mechanical stimulation, nutrient exposure, or microbial metabolites trigger EC cells to release serotonin (5-HT) (see Figure 3). This serotonin then activates 5-HT_3_ receptors on vagal nerve terminals, increasing afferent signaling [1,32]. The resulting multimodal sensory input converges in the nucleus of the solitary tract (NTS), which projects to downstream regions that regulate appetite (e.g., lateral parabrachial nucleus and hypothalamus), mediate stress and emotional responses (e.g., central amygdala and bed nucleus of the stria terminalis), and support cognitive functions (e.g., hippocampus via locus coeruleus and hypothalamic relays). Furthermore, gut microbial metabolites, including SCFAs [97], secondary bile acids, and tryptophan-derived indoles, have been shown to modulate vagal activity either directly (e.g., via FFAR3 receptors on vagal afferents) [32,66,98] or indirectly through hormonal intermediates such as glucagon-like peptide-1 (GLP-1), cholecystokinin [99] (CCK), or peptide YY (PYY) [89]. This dual mechanism enables the microbiota to dynamically influence brainstem and limbic activity in response to changes in gut microbial state [1,32,100]. Overall, the vagus nerve acts as a rapid and adaptable conduit through which intestinal signals, including nutrient cues, microbial metabolites, and mechanical stimuli, can influence brainstem activity, limbic processing, and higher-order cognitive functions.

By integrating multimodal input from EEC and EC cells, vagal afferents (see Figure 3) enable the central nervous system to continuously monitor and respond to the dynamic state of the gut. This bidirectional communication underscores the gut’s capacity to shape behavior, affect, and cognition in a context-dependent manner, highlighting the vagus nerve as a central mediator of gut–brain interactions.

Critically, these vagal sensory pathways are not fully static but undergo significant refinement across development. During adolescence, a period marked by widespread neural remodeling, hormonal fluctuations, and behavioral maturation, the structure, connectivity, and functional responsiveness of vagal afferents are hypothesized to be particularly plastic [37,83]. This developmental window may therefore represent a period of heightened sensitivity to gut-derived signals, with potential implications for appetite regulation, emotional processing, and cognitive function [101]. Exploring how vagal circuits mature during adolescence provides a framework for understanding age-specific gut–brain interactions and identifying potential periods of vulnerability or resilience in neurobehavioral development.

Although studies directly characterizing vagal afferent maturation during adolescence remain limited, emerging preclinical evidence suggests that this developmental period involves significant refinement of gut–brain sensory communication. The gut–brain axis undergoes dynamic restructuring in adolescence: as the gut microbiota diversifies and the enteric nervous system (ENS) continues to mature, afferent connections between the GI tract and brain may also undergo pruning, remodeling, or functional tuning [2,8]. Indeed, recent reviews emphasize that adolescence constitutes a tentative “sensitive window” for the microbiota–gut–brain (MGB) axis, during which ENS maturation, hormonal shifts, and microbial changes interact to potentially influence the trajectory of neural development [2] (see Figure 3).

In this context, vagal afferent neurons may be susceptible to environmental changes; for example, inflammatory insults, antibiotic exposure, or drastic dietary changes could disrupt the normal maturation of gut–brain signaling pathways [2]. Given that vagal tone has been proposed as a physiological read-out of vagal integrity and gut–brain communication, altered or insufficient vagal maturation during adolescence could theoretically increase vulnerability to dysregulated stress responses or disrupted behavioral outcomes [2,48].

It is critical to note that while recent experimental data in animal models provide compelling evidence that intact vagal signaling is essential for hippocampal plasticity and emotional behavior, these findings rely heavily on absolute preclinical disruptions such as surgical vagotomy or complete microbiota depletion in rodents [102]. Because direct experimental vagal manipulation is neither feasible nor ethical in human youth, these pathways remain associated in human populations. Changes to vagal signaling during adolescence due to microbiome disruption, inflammation, or environmental stressors are highly likely to modulate cognitive and affective development, but direct mechanical causality in human adolescents has yet to be empirically established.

### 4.1. Gut Microbiome and Mesocorticolimbic Reward Circuits

#### 4.1.1. Preclinical Findings in Adult Animals and Developmental Hypotheses Extrapolated to Adolescence

Much of our current understanding regarding the microbiome’s role in mesocorticolimbic dopamine function and reward-related behavior stems from studies conducted on adult animals or general rodent models. In adult rodents, depletion of the gut microbiome—either via germ-free (GF) rearing or broad-spectrum antibiotic treatment—attenuates psychomotor activation and conditioned place preference (CPP) for substances like cocaine, effects that can be reversed by microbiome reconstitution [103]. Similarly, adult GF and antibiotic-treated animals show altered basal dopamine turnover, changes in the electrophysiological excitability of dopamine-releasing ventral tegmental area (VTA) neurons, and disrupted reward-seeking and novelty-seeking behaviors [62]. Adult rats treated with antibiotics also demonstrate increased motivation for low-dose rewards and enhanced cue-induced seeking behavior following prolonged abstinence, which can be rescued by replenishing microbially derived SCFAs [100].

Furthermore, adult models demonstrate that high-fat–derived microbiota can reduce extracellular dopamine metabolite (DOPAC) levels in the nucleus accumbens (NAc), decrease incentive-driven feeding, and downregulate dopamine D2 receptors—a hallmark of altered reward sensitivity that was selectively reversed via gut-to-brain vagal deafferentation [69]. General models also confirm that the adult microbiota influences basic dopamine biosynthesis, turnover, and the expression of essential synaptic proteins like PSD-95 and synaptophysin [104].

Biomedical researchers frequently extrapolate these adult findings to formulate developmental hypotheses for the adolescent period. Because the mesocorticolimbic dopamine pathways undergo protracted maturation, structural refinement, and peak plasticity during youth [39], it is hypothesized that perturbations such as diet-induced microbiota shifts or antibiotic treatment may derail the normal developmental trajectory of dopamine signaling. While these adult data suggest a potential mechanism by which early environmental factors shape long-term behavioral trajectories, susceptibility to substance use disorders, or reward dysregulation, direct empirical confirmation within adolescent cohorts remains a crucial missing piece of the literature.

#### 4.1.2. Empirical Evidence Observed Specifically During Adolescence

In contrast to adult models, empirical evidence collected specifically during the adolescent developmental window highlights a unique vulnerability. Emerging evidence suggests that during adolescence, gut-derived signals actively modulate the pronounced remodeling of prefrontal-striatal projections and heightened dopaminergic sensitivity characteristic of this age range [59,70]. Preclinical studies restricted to adolescent animals demonstrate that antibiotic-induced depletion of the microbiota specifically dampens dopamine release in the nucleus accumbens (NAc) and disrupts ongoing experience-dependent reward refinement; notably, these adolescent-specific effects can be rescued by targeted recolonization with (SCFA)-producing microbial communities [52,69,100,105].

These precise adolescent cohorts demonstrate that microbial metabolites (e.g., SCFAs, tryptophan, and tyrosine derivatives) alter the excitability of VTA neurons and regulate dopamine receptor expression (specifically D1- and D2-like receptors) at a time when these systems are undergoing natural structural pruning [70]. This indicates that the adolescent reward system is distinctly sensitive to microbial shifts compared to its adult counterpart, as microbial dysbiosis during this brief window can permanently alter how rewarding stimuli are perceived, valued, and integrated into long-term goal-directed behaviors.

#### 4.1.3. Role of Microbial Metabolites and Vagal Pathways in Reward Circuitry

In line with this, rodent models show that germ-free rearing or microbiota depletion leads to exaggerated dopamine turnover, altered excitability of ventral tegmental area (VTA) neurons, and enhanced locomotor and novelty-seeking behaviors phenotypes consistent with heightened reward sensitivity [62,69,100]; Likewise, depletion of microbiota via broad-spectrum antibiotics reduces dopamine release in the nucleus accumbens (NAc) and dampens reward-seeking behavior; importantly, these effects can be rescued by recolonization with short-chain fatty-acid (SCFA)-producing microbial communities [55,88,105]. These data support the idea that microbial metabolites contribute to shaping dopamine system function, particularly during periods of developmental plasticity (e.g., adolescence).

#### 4.1.4. Implications for Addiction Vulnerability

The gut microbiome can modulate dopamine receptor expression and synaptic plasticity within key reward circuits such as the nucleus accumbens (NAc) and prefrontal cortex (PFC) [69]. In rodent models, manipulation of microbiota composition alters dopaminergic transmission: for example, a high-fat–derived microbiota was shown to reduce extracellular dopamine metabolite (DOPAC) levels in the NAc [69] and decrease incentive-driven feeding; these changes were accompanied by reduced expression of dopamine D2 receptors, a hallmark of altered reward sensitivity, and were reversed by selectively deafferenting gut-to-brain vagal pathways.

**Figure 4:**
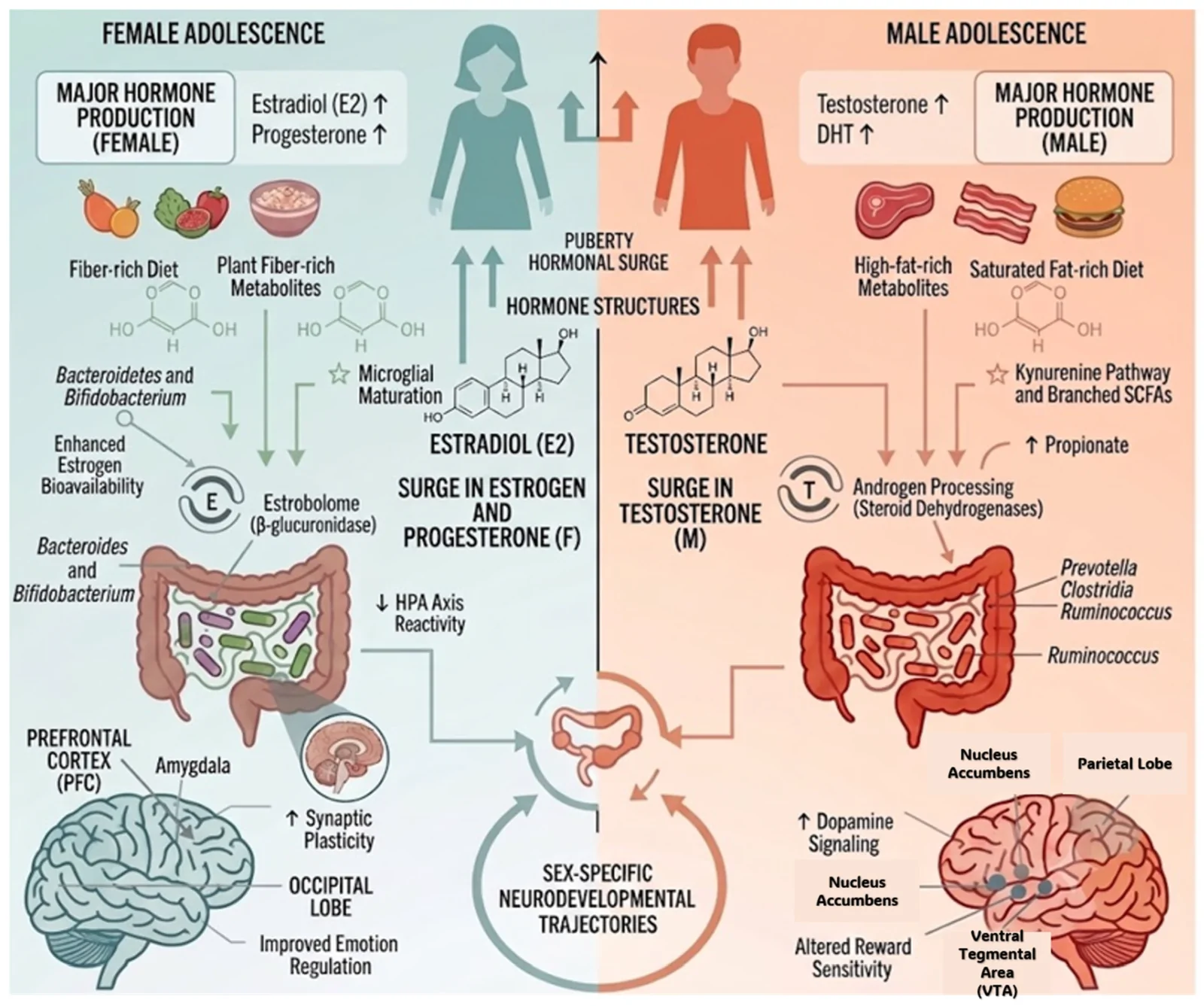
The Sex-Specific Gut–Brain–Microbiome Axis During Adolescent Neurodevelopment. Adolescence is a neurobiological inflection point where pubertal endocrine surges—primarily estradiol in females and testosterone in males—intersect with sex-differentiated dietary patterns to drive divergent microbial trajectories. The bidirectional relationship between sex steroids and microbial metabolism is emphasized, highlighting that hormones regulate microbial composition while microbial enzymatic activity influences steroid bioavailability and downstream neuroendocrine signaling. In females, a fiber-rich diet and high estrogen levels promote the estrobolome, characterized by Bacteroides-mediated beta-glucuronidase activity that increases systemic estrogen bioavailability and the production of butyrate and serotonin precursors. These signals facilitate synaptic plasticity in the prefrontal cortex and regulate the HPA axis, influencing vulnerability to internalizing disorders like anxiety and depression. Conversely, in males, diets higher in saturated fats and rising androgens select for androgen-processing taxa such as Clostridia and Prevotella, which enhance propionate production and kynurenine pathway flux. These metabolites calibrate mesolimbic dopaminergic signaling and reward sensitivity within the ventral tegmental area and nucleus accumbens, shaping male-biased risks for externalizing behaviors and substance use. Together, these pathways position the microbiome as a critical mediator of sexually dimorphic brain maturation and mental health risk. The conceptual design, scientific pathways, and thematic frameworks of the figures were conceived and developed entirely by the authors. Artificial intelligence generation tools (Midjourney/DALL-E) were utilized solely to assist with graphic rendering, layout execution, and visual styling. All text, scientific labels, and pathways were manually reviewed and verified by the authors.

More broadly, evidence from germ-free and antibiotic-mediated dysbiosis models supports a fundamental role for gut microbes in dopamine metabolism and receptor regulation [104]. A recent review summarized data showing that the microbiota may influence both dopamine biosynthesis and turnover, as well as the expression of synaptic proteins (e.g., PSD-95, synaptophysin) and dopamine receptor subtypes thereby shaping the plasticity of mesolimbic and striatal circuits. Because synaptic plasticity and receptor expression in the NAc and PFC underlie reward learning, motivation, and reinforcement sensitivity, these microbiome-driven molecular changes have significant behavioral implications. Perturbations such as diet-induced microbiota shifts, antibiotic treatment, or other sources of dysbiosis can therefore alter reward valuation, affect sensitivity to natural or drug rewards, and shape long-term behavioral trajectories, including susceptibility to addictive behaviors or maladaptive reward seeking. These findings collectively highlight the gut microbiome as a major regulator of reward-related neural plasticity, not only influencing baseline dopamine signaling but also the structural and molecular architecture of reward circuits. Such microbiome–dopamine–behavior interactions may thus represent critical mechanisms by which early environmental factors (e.g., diet and changes in microbiome composition) influence vulnerability to substance use disorders, maladaptive motivation, or reward dysregulation across development (see Figure 4).

### 4.2. Endocrine and Immune Interfaces

The HPA axis is regulated by microbial input, with germ-free or antibiotic-treated rodents showing exaggerated stress responses. This crosstalk is emerging as a key mechanism linking gut microbiota to adolescent brain development and emotional behavior. The gut microbiome plays a pivotal role in regulating endocrine and immune pathways that influence brain function during adolescence. These effects occur through bidirectional signaling along the gut–brain axis, affecting stress and emotional processing. Microbial composition shapes HPA axis activity. The gut microbiota also shapes immune signaling, which can feed back to HPA axis regulation. Dysbiosis can elevate systemic and local pro-inflammatory cytokines [106], disrupt the intestinal barrier, and alter host neuroendocrine signaling, all of which may contribute to maladaptive HPA axis responses under stress.

Germ-free and antibiotic-treated mouse models have been used to target the mechanisms by which microbial communities regulate neurodevelopment. These models allow researchers to manipulate microbiota at various stages of development, allowing study of critical windows which the gut–brain interactions may be particularly impactful on stress responsiveness and cognitive function. GF mice exhibit profound alterations in anxiety-like behavior, elevated corticosterone levels, reduced hippocampal BDNF levels, and altered synaptic plasticity compared with conventionally housed control mice [62]. These observations suggest that the presence of a normal microbiota exerts a calibrating effect on the HPA axis reactivity. Germ-free and antibiotic-treated rodents display exaggerated corticosterone responses to stress and impaired glucocorticoid feedback regulation [37].

In parallel, the microbiome modulates immune development by tuning microglial maturation, cytokine signaling, and peripheral immune tone, all of which undergo major transitions during adolescence [9,48]. These changes are critical for establishing neural circuits that regulate stress and emotional behavior. These endocrine–immune pathways provide a slower but highly influential route through which microbial signals regulate emotional behavior, stress responsivity, and neuroplasticity during this sensitive developmental window. This highlights the potential of early microbial interventions to support adolescent mental health.

During adolescence, the HPA axis and CNS undergo rapid maturation, increasing sensitivity to stressors. Heightened anxiety and depression in adolescents have been linked to alterations in the gut microbiota, or dysbiosis. These disruptions may be particularly relevant in adolescents exposed to opioids [43,107,108] (see Figure 3).

#### Importance of the Microbiome in Stress Responses and Cognition

The impact of the gut microbiome extends beyond stress responses; it also plays a critical role in shaping cognitive functions during adolescence. A substantial body of evidence suggests that the gut microbiome contributes not only to modulating stress-axis reactivity but also to shaping cognitive functions and neuroplasticity, particularly through its effects on hippocampal structure and function. The mammalian hippocampus is a central brain region to learning, memory, and stress adaptation [80] and it appears especially sensitive to signals derived from the intestinal microbiota [83]. Indeed, several preclinical studies demonstrate that alterations in microbiota composition can impair hippocampal neurogenesis, disrupt neurotrophins expression, and compromise cognitive performance and stress resilience [12,109].

In rodent models, depletion or dysregulation of gut microbiota, whether via germ-free rearing, antibiotic treatment, or stress-induced dysbiosis, is associated with reduced proliferation and survival of newly generated neurons in the hippocampal dentate gyrus, as indicated by decreased markers such as doublecortin (DCX) or BrdU incorporation [15,110].

Crucially, it must be emphasized that direct measurement of hippocampal neurogenesis using cellular markers like DCX or BrdU in living human adolescents is not currently technologically feasible. Consequently, these insights regarding the absolute suppression and restoration of neurogenesis originate entirely from experimental animal studies. While these findings provide a compelling mechanistic model for how gut dysbiosis impairs hippocampal neuroplasticity and decreases brain-derived neurotrophic factor (BDNF) [111], their translational implications for human teenagers must be evaluated with caution. In human cohorts, these cellular phenomena can only be inferred through indirect proxies like structural neuroimaging or cognitive testing. Mechanistic studies implicate several overlapping pathways by which the microbiome may exert influence on hippocampal neurogenesis and cognitive function. Microbial metabolites, such as SCFAs and indole derivatives, are candidate mediators that can cross the intestinal barrier and influence host cells, including immune cells and neural progenitors [88,111].

Through immune signaling, the microbiome can regulate microglial activation and cytokine release [62] in the brain, processes that in turn affect synaptic remodeling, neuronal survival, and neurogenesis. In parallel, the microbiota–gut–brain axis includes neuroendocrine signaling, gut microbial composition influences stress-related endocrine pathways (e.g., glucocorticoid release via the HPA axis), which can suppress hippocampal neurogenesis when chronically elevated [92,106,111]. Recent experimental work underscores the functional relevance of these interactions for stress resilience and cognitive outcomes. In a mouse model of chronic unpredictable mild stress (CUMS), transplantation of fecal microbiota from stress-resilient donors characterized by relatively higher abundance of genera such as *Lactobacillus*, *Bifidobacterium*, and *Romboutsia* restored hippocampal neurogenesis and ameliorated behavioral deficits associated with depression and anxiety [103,104,112]. Critically, pharmacological blockade of neurogenesis prevented the beneficial effects of microbiota transplantation on stress-induced behavioral impairment [83], providing causal evidence that microbiome-dependent restoration of neurogenesis mediates improved stress resilience. These findings collectively suggest that gut dysbiosis whether from antibiotics, stress, poor diet, or other environmental insults may impair hippocampal neurogenesis and neuroplasticity, thereby increasing susceptibility to cognitive deficits, maladaptive stress responses, and psychiatric disorders [62,92,111]. Given the dynamic development of both the microbiome and the central nervous system during adolescence, this period may represent a particularly vulnerable window wherein disruption of microbial composition could have especially profound and long-lasting consequences for brain function. Thus, early-life or adolescence-targeted interventions should aim at maintaining or restoring healthy gut microbial communities.

Nevertheless, important limitations remain. Much of the evidence arises from animal studies; human data linking gut microbiome composition, hippocampal neurogenesis (or proxies thereof), and cognitive/emotional outcomes remain scarce. Moreover, the specific microbial taxa, metabolites, and signaling pathways responsible for beneficial versus detrimental effects remain incompletely defined. Future research should aim to characterize these mechanisms with high taxonomic and functional resolution, and to test microbiome-targeted interventions longitudinally during critical developmental windows. In conclusion, the microbiome’s influence on neurogenesis, neurotrophin expression, immune modulation, and neuroendocrine signaling collectively position it as a potent regulator of hippocampal-dependent cognition and stress adaptability. This underscores the importance of gut microbial health for brain resilience, especially during periods of neurodevelopmental vulnerability such as adolescence.

### 4.3. Microbial Metabolites: Short-Chain Fatty Acids, Tryptophan Derivatives, Bile Acids, and Their Neural Targets

Microbial metabolites exert further influence on adolescent brain maturation by shaping neurotransmitter synthesis, synaptic refinement, and myelination. SCFAs such as acetate promote oligodendrocyte proliferation and myelin formation, supporting the maturation of prefrontal circuits that continue to refine into early adulthood. Indole and kynurenine pathway metabolites regulate microglial function and synaptic pruning, processes essential for sculpting adolescent cortical circuitry. Bile-acid derivatives and tryptophan metabolites also impact monoaminergic neurotransmission, influencing serotonin and dopamine availability. Together, these molecular mediators form a diverse biochemical interface through which the microbiome can shape long-term trajectories of cognitive, emotional, and reward-related development.

The intestinal microbiome generates a complex molecular milieu composed of SCFAs, secondary bile acids, indole derivatives, tryptamine, lactate, and lipopolysaccharide (LPS) fragments, which interact with enteroendocrine cells and vagal afferent terminals to modulate enteric and central neural activity.

Acetate, propionate, and butyrate are SCFAs that are generated by microbial fermentation of dietary fiber, They activate free fatty acid receptors (FFAR2/3) [97] expressed on enteroendocrine and immune cells. These interactions regulate vagal firing indirectly via hormone release (GLP-1, PYY, CCK) and directly through vagal FFAR3 signaling [97,113]. SCFAs also cross the blood–brain barrier, modulate microglial maturation and influence NTS and limbic excitability [61,99].

Microbial tryptophan metabolism produces indole, tryptamine, and kynurenine, which are pathway intermediates that affect the vagal signaling and limbic function. Tryptamine stimulates 5-HT receptors on EC cells, promoting 5-HT release, which increases vagal activity. Indoles engage aryl hydrocarbon receptors (AHRs) in epithelial and immune cells, regulating cytokine expression and gut permeability, which indirectly modulates vagal inflammatory tone [114].

Bacterial bile-salt hydrolase activity converts primary bile acids into secondary bile acids, including deoxycholic acid and lithocholic acid, which activate Takeda G protein-coupled receptor 5 (TGR5) expressed on enterochromaffin (EC) cells and enteric neurons. Activation of these receptors stimulates vagal signaling pathways that influence metabolic regulation, gastrointestinal signaling, and hedonic or reward-related responses [113,115].

## 5. Gut–Brain–Microbiome Sex Differences

Adolescence represents a developmental epoch during which interactions among sex hormones, microbial communities, and neural circuits undergo rapid transformation. Accumulating evidence suggests that these processes unfold in sex-specific trajectories, with important implications for mental health vulnerability. Here, we review how pubertal endocrine surges shape gut microbial diversification, how microbial enzymes influence steroid metabolism and neuroendocrine signaling, how diet and environmental factors modulate these pathways, and how these interactions contribute to sex-biased risk for anxiety, depression, and neurodevelopmental disorders. Integrating human and animal findings, we propose a mechanistic framework positioning the microbiome as a key mediator of sexually dimorphic neurodevelopment during adolescence (see Figure 4).

Adolescence represents a pivotal period marked by extensive neurodevelopmental, hormonal, and microbial changes, with sex-specific factors playing a crucial role in shaping the MGB axis. Although the bidirectional communication between the gut microbiome and brain—via vagal nerve innervation, neuroendocrine, and neuroimmune pathways—is well established, the influence of sex and hormonal modulators on this axis remains relatively understudied, particularly during adolescence, a phase characterized by heightened neuroplasticity and hormonal surges. Recent work increasingly underscores that adolescence is not merely a continuation of childhood maturation but a neurobiological inflection point marked by sharp divergence in male and female developmental trajectories. These divergences unfold simultaneously across endocrine signaling, microbiome composition, immune activation, and neural circuit refinement [2,116]. Pubertal hormones, particularly estradiol and testosterone, reorganize cortical and limbic pathways in sex-dependent ways [18] while microbial communities become increasingly specialized and metabolically complex [4]. This section synthesizes these emerging threads to examine how sex-specific hormonal dynamics intersect with microbial maturation to shape neurodevelopmental outcomes during adolescence. We organize the following information into four conceptual domains aligned with mechanistic pathways supported by human and animal findings.

### 5.1. Pubertal Hormonal Surges Drive Sex-Dependent Microbial Diversification

#### 5.1.1. Puberty as a Microbiome-Shaping Event

Puberty represents one of the most dramatic endocrine transformations in the human lifespan, characterized by up to ten-fold increases in circulating estradiol, progesterone, and testosterone [117]. These steroid hormones exert strong selective pressures on microbial niches, affecting mucosal secretions, intestinal permeability, nutrient availability, and immune activation.

#### 5.1.2. Human Cohort vs. Rodent Distinction

Longitudinal clinical data tracking of human adolescent cohorts reveal that microbial alpha-diversity increases significantly throughout pubertal maturation, though the trajectory and composition diverge sharply by sex [85,86,105] However, because direct mechanical manipulation of systemic endocrine levels is restricted to preclinical designs, our specific taxonomic insight into this selective pressure comes from murine models. These rodent studies demonstrate that rising host estrogens selectively enrich *Bacteroides* and *Akkermansia* species (taxa containing pathways capable of estrogen processing), whereas rising androgens in male mice select for *Prevotella* and certain *Clostridia* strains [20,86,118] (see Figure 4).

#### 5.1.3. Neurodevelopmental Implications of Sex-Specific Microbial Maturation

Because microbial shifts differ between males and females, the metabolite landscape influencing the adolescent brain is also hypothesized to be highly sexually dimorphic [101,118]. Pubertal sex steroids and the gut microbiome engage in a bidirectional regulatory relationship that may contribute to sex-specific trajectories of adolescent neurodevelopment. Rising concentrations of estradiol, progesterone, and testosterone during puberty influence microbial ecology by altering intestinal physiology, including epithelial barrier function, immune tone, nutrient availability, and bile acid metabolism. Conversely, microbial communities regulate circulating steroid availability through enzymatic pathways collectively referred to as the estrobolome, including bacterial β-glucuronidase activity that modulates estrogen deconjugation and enterohepatic recycling. These interactions create a dynamic endocrine–microbial feedback system in which microbiota composition can influence hormone signaling, while hormonal maturation shapes microbial community structure. Importantly, these processes occur during adolescence, when both endocrine systems and neural circuits involved in emotion regulation, reward processing, and stress responsivity undergo extensive remodeling. Therefore, sex-dependent microbiome maturation may represent an important biological mechanism contributing to divergent vulnerability or resilience to neuropsychiatric and metabolic outcomes across males and females.

#### 5.1.4. Preclinical Animal Findings

In rodent models, the functional relevance of these shifts has been confirmed through direct endocrine manipulation. Experimental rodent studies show that male and female gut microbiomes respond divergently to gonadectomy, hormone replacement, and isolation stress [20,101]. For instance, in male germ-free (GF) F344 rats, absence of commensal microbiota causes a severe phenotypic characterized by increased anxiety-like behavior, amplified HPA-axis responses to acute stressors, and disrupted dopamine turnover in frontolimbic regions [72]. Complementary germ-free mouse models confirm sex-dependent variations in central serotonergic pathways, demonstrating that absent microbial colonization yields highly dimorphic behavior phenotypes depending on the host genetic strain and sex [1,87]. These models establish that circulating microbial metabolites (including SCFAs like male-biased valerate or female-biased butyrate precursors) modulate cellular milestones like neuroplasticity, microglial synaptic pruning, and myelination during the peri-pubertal window [2].

#### 5.1.5. Human Adolescent Evidence and Hypotheses

In human populations, direct mechanical verification of these central tissue changes is unavailable. Evidence from human adolescent cohorts is instead associative, linking cross-sectional stool taxonomy and serum metabolomic profiles to behavioral questionnaires. Human data confirms that sex differences in gut composition mirror divergent emotional trajectories during puberty, supporting the hypothesis that the human microbiome acts as an upstream regulator of sex-biased neurodevelopment [119,120]. However, causal links tracing specific human taxa directly to altered prefrontal receptor expression remain speculative.

### 5.2. Microbial Enzymes and Steroid Metabolism: The Estrobolome and Androgen-Processing Microbiota

Microbial steroid-metabolizing enzymes within the gut microbiome shape peripheral hormone pools, thereby influencing sexually dimorphic trajectories of brain development across adolescence. Diverse bacterial intent-groups express beta-glucuronidases, sulfatases, and reductases that collectively constitute a broader “endobolome,” transforming sex steroids into locally and systemically bioactive forms [20,121]. In human adolescent females, sequencing studies confirm that the activity of the estrobolome increases substantially during puberty, corresponding with natural expansions of estrogen-responsive taxa such as *Bacteroides, Ruminococcus,* and *Eggerthella* [86,118]. By deconjugating estrogens via beta-glucuronidase pathways, these microbes prevent hormone excretion, thereby modulating systemic estradiol bioavailability [20]. While human data confirms correlations between estrobolome composition and circulating estradiol, the direct downstream neural consequences are characterized primarily through preclinical animal models. Experimental work in rodents shows that alterations in estrogen tone directly modify synaptic proteins such as PSD-95 and remodel dendritic spines within the hippocampus and prefrontal cortex [18,122]. Furthermore, preclinical dysbiosis models induced by antibiotics or chronic stress show blunted estrogen signaling, HPA axis disinhibition, and heightened anxiety- or depression-like phenotypes [50]. While these multi-level interactions position the estrobolome as a major vulnerability factor for female-biased internalizing psychopathology, direct human mechanistic confirmation linking gut beta-glucuronidase concentrations to cortical synaptic density during female adolescence is lacking and must be viewed as an ongoing hypothesis.

#### 5.2.1. Androgen-Modifying Microbiota and Male Brain Pathways

Parallel to the estrobolome, an “androgen-modifying” gut microbiota participates in the intestinal deglucuronidation and transformation of testosterone and dihydrotestosterone (DHT). Preclinical data demonstrates that specific taxa, including Clostridium scindens and Butyricicoccus desmolans, express steroid dehydrogenases that convert sex steroids into bioactive derivatives capable of re-entering systemic circulation [123]. Pubertal rodent models show that rising testicular androgens organize male-typical gut profiles that persist into adulthood; castration during this window eliminates these differences, while testosterone replacement rescues them, showing that pubertal androgens function as host-selection pressures [124,125].

##### Human vs. Animal Causal Boundaries

In human populations, observational data confirms that male gut microbial composition correlates positively with serum testosterone levels and downstream androgen metabolites, with higher-testosterone males exhibiting distinct enrichment of these steroid-metabolizing strains. However, causal directionality remains largely delineated by animal experiments. Preclinical microbiota transfer studies show that colonizing female mice with adult male microbiota successfully elevates systemic testosterone and masculinizes their peripheral immune profiles—an effect halted by androgen receptor antagonism [122]. Animal data further links this androgen–microbiota feedback loop to changes in dopaminergic release and receptor expression within the VTA and nucleus accumbens, which modulates phenotypic aggression and reward sensitivity [72]. In human adolescents, while these pathways strongly implicate a bidirectional loop where microbial enzymes tune male androgen tone and influence externalizing behaviors, human evidence is presently correlative and relies heavily on these preclinical models for its mechanistic framework [124].

### 5.3. Interactions Between Diet, the Gut Microbiome, and Hormonal Regulation of Neuroendocrine Signaling

#### 5.3.1. Diet as a Sex-Dependent Modulator of Microbial Ecosystems

Adolescence is a developmental stage in which diet becomes a primary ecological force shaping the gut microbiome, and emerging evidence indicates that these effects are deeply sex-dependent. Longitudinal human adolescent cohort studies show that dietary quality begins to diverge between the sexes as early as age 10–12, running parallel to rising gonadal hormone levels [117,119,122]. Human data show that females demonstrate earlier increases in fiber, fruit, and phytoestrogen intake—dietary components known to enrich *Bifidobacterium* and *Lachnospiraceae* [1,126,127]—whereas human males show higher intake of saturated fats and red meat, which correlates with bile-tolerant taxa like *Alistipes* [82,95].

This sex-differentiated pattern is highly apparent when examining essential nutrients like omega-3 fatty acids. Stool and serum testing from human adolescent cohorts reveal that females with low omega-3 levels exhibit heightened depressive symptoms and altered microbial alpha-diversity [125,128], whereas human males appear more sensitive to omega-3 deficiencies via markers of impulsivity. To investigate the underlying mechanisms of these findings, researchers rely on rodent models. Peri-pubertal rodent studies demonstrate that experimental omega-3 deficiencies induce significantly harsher reductions in microbial diversity in females than in males [22]. Furthermore, direct histological analysis in these peripubertal animal designs reveals that omega-3 supplementation actively downregulates microglial activation and HPA-axis sensitivity in females, but preferentially upregulates dopaminergic signaling metrics in males [22]. These dual tracks reinforce the necessity of validating preclinical dietary interventions [60] through sex-specific human clinical trials.

#### 5.3.2. Microbial Metabolites Link Dietary Inputs to Neural Signaling

Microbial metabolites are key biochemical mediators linking these sex-specific diet-microbiome interactions to neurodevelopmental outcomes (see Figure 4). Human adolescent profiling confirms that males and females possess distinct microbiome-encoded metabolic profiles, showing differential enrichment of genes governing SCFA production and aromatic amino acid metabolism [118,120] Observational cohorts confirm that peripheral kynurenine-to-tryptophan ratios are significantly higher in human males than females from ages 12–18, reflecting a distinct metabolic shift during male pubertal maturation [98].

The downstream neurodevelopmental effects of these metabolic variances are largely derived from mechanistic hypotheses based on preclinical tissue studies. In animal models, fiber fermentation yields SCFAs (acetate, propionate, butyrate) (see Figure 4) that cross the blood–brain barrier to calibrate microglial maturation and modulate frontolimbic neuroinflammation [1] Similarly, animal data demonstrates that *Bifidobacterium* and *Lactobacillus* (strains enriched in adolescent human females) convert tryptophan into protective indoles that modulate central 5-HT receptor expression, whereas male-biased taxa drive tryptophan down the kynurenine pathway, generating quinolinic acid that impacts hippocampal glutamatergic transmission [66,129]. While these distinct biochemical pathways provide an elegant framework for explaining why internalizing disorders dominate female adolescence and reward-processing conditions dominate male adolescence, clinical researchers must remain cautious. Direct empirical evidence proving that human adolescent behavioral changes are caused by these specific metabolite-receptor interactions in the brain remains unavailable.

## 6. Conclusions

Adolescence emerges as a developmental inflection point in which sex hormones, microbial consortia, and neural circuit remodeling accelerate simultaneously, creating a biologically sensitive period with long-lasting implications for mental health. Across the evidence reviewed, several convergent themes stand out. First, pubertal endocrine shifts provide strong selective pressures on the intestinal ecosystem, promoting sex-dependent microbial diversification that reshapes metabolic and immunological outputs.

Second, microbial enzymatic systems including the estrobolome and androgen-metabolizing taxa are hypothesized to participate in steroid biotransformation, potentially modifying the systemic hormonal cues that guide synaptic pruning, cortical maturation, and limbic circuit reorganization. Third, diet and lifestyle factors interact with these biological transitions, potentially amplifying sex differences [124,130] via nutrient-responsive microbes, short-chain fatty acid production, and tryptophan/kynurenine pathway regulation. Finally, these integrated processes yield distinct metabolite landscapes in males and females, offering a compelling conceptual framework to help explain sex biases in internalizing symptoms, reward-related behaviors, and neurodevelopmental risk during adolescence.

Rather than viewing the microbiome merely as a passive downstream consequence of pubertal physiology, the accumulated findings allow us to hypothesize that it functions as an active participant—one capable of modulating steroid availability, immune tone, and neurotransmitter precursor pools at precisely the moment when the brain is most plastic. However, it is vital to emphasize that while this active mechanistic role is strongly supported by tightly controlled preclinical animal models, direct causal evidence in human adolescent populations remains limited.

Although substantial progress has been made, several limitations remain. Current evidence is derived from heterogeneous experimental approaches, including animal models, cross-sectional human studies, and observational cohorts. While animal studies provide important mechanistic insights, differences in microbial composition, dietary patterns, developmental timing, and environmental exposures complicate direct translation to human populations. Human studies are additionally limited by variability in sequencing platforms, incomplete longitudinal sampling, challenges in controlling diet and medication exposure, and a persistent difficulty in establishing clear causal relationships between microbial changes and neurodevelopmental outcomes.

Consequently, many of the highly detailed biochemical mechanisms proposed in current literature have yet to be rigorously validated in living human teenagers. Future investigations must prioritize large-scale, prospective longitudinal adolescent cohorts integrating microbiome sequencing, metabolomics, endocrine profiling, immune markers, and neurobehavioral assessments to track these configurations over time. Multi-omics approaches and standardized developmental frameworks will be essential for identifying true microbial signatures associated with resilience or vulnerability. Furthermore, rigorous intervention studies evaluating diet modification, exercise, probiotics, prebiotics, and targeted microbial metabolites are strictly required to move past correlation and definitively determine whether targeted modulation of the microbiome can improve adolescent brain health.

Collectively, evidence reviewed here supports and extends previous research demonstrating that the microbiome–gut–brain axis represents a conserved developmental system influenced by microbial metabolites, immune signaling, endocrine regulation, and neural plasticity. Compared with earlier studies focused primarily on infancy or adulthood, emerging adolescent research highlights this developmental stage as a unique period of microbiome remodeling and neuroendocrine sensitivity. Properly validating these interactions through human-centered translational research may ultimately provide new, evidence-based opportunities for preventive strategies targeting mental health, metabolic disorders, and substance-use vulnerability.

## Data Availability

No new data were created or analyzed in this study. Data sharing is not applicable to this article.

## Supplementary Materials

The following supporting information can be downloaded at: https://www.mdpi.com/article/10.3390/nu18172891/s1, Table S1. Summary of Key Evidence Supporting Microbiome–Gut–Brain Axis Regulation During Adolescence.
