# Supplementary material for: The Adolescent Microbiome–Gut–Brain Axis: Development, Mechanisms, and Translational Perspectives
Source: Nutrients. 2026 Sep 3;18(17):2891. doi: 10.3390/nu18172891 (PMC13567016; doi:10.3390/nu18172891)
Supplement: Supplementary file 1 [file nutrients-18-02891-s001.zip › nutrients-4366093-supplementary.pdf]

Table S1. Summary of Key Evidence Supporting Microbiome–Gut–Brain Axis Regulation During Adolescence

Evidence category	Experimental approach/model	Major findings relevant to adolescent neurodevelopment	Proposed mechanisms linking microbiota to brain function	Representative references
**Microbiota regulation of brain development and behavior**	Germ-free (GF) animals, microbial colonization studies, and microbiota manipulation models	Absence or disruption of microbiota alters stress responsivity, anxiety-like behaviors, neurodevelopmental trajectories, and expression of neuroplasticity-related genes. Restoration of microbial communities during developmental windows can partially normalize behavioral phenotypes.	HPA-axis regulation, altered neurotransmitter signaling, immune maturation, neurotrophic factors including BDNF, and microbial metabolite signaling	Sudo et al., 2004; Diaz Heijtz et al., 2011; Crumeyrolle-Arias et al., 2014; Cryan et al., 2019
**Adolescent microbiome as a critical developmental window**	Developmental microbiome profiling across life stages; adolescent animal and human studies	Adolescence represents a period of simultaneous microbial maturation, pubertal hormone changes, immune remodeling, and neural circuit refinement, suggesting increased sensitivity to microbiome perturbations.	Interaction between microbial metabolites, synaptic maturation, immune signaling, endocrine regulation, and adolescent brain plasticity	Borre et al., 2014; Flannery et al., 2019; Carson et al., 2023; Cryan, 2025

**Microbiome disruption and neurobehavioral outcomes**	Antibiotic-induced microbiome depletion and adolescent microbiota perturbation models	Microbiome depletion during adolescence produces persistent behavioral alterations, including anxiety-like behaviors, cognitive changes, and altered stress responses.	Microglial activation, neuroinflammation, altered hippocampal neurogenesis, neurotransmitter metabolism, and immune-mediated signaling	Möhle et al., 2016; Lach et al., 2020; Cook and Prinz, 2022; Fishbein et al., 2023
**Microbial regulation of neurotrophic pathways**	Microbiota manipulation combined with molecular analysis of brain tissue	Alterations in gut microbial communities modify central BDNF expression and neuronal signaling pathways associated with cognition, emotional regulation, and synaptic plasticity.	BDNF signaling, hippocampal plasticity, serotonin pathways, immune modulation, and microbial-derived metabolites	Bercik et al., 2011; Dash et al., 2022; Cryan et al., 2019
**Microbial metabolites as mediators of brain function**	Metabolomics, microbial functional profiling, SCFA analysis, and dietary interventions	Microbial-derived metabolites including short-chain fatty acids (SCFAs), bile acids, and tryptophan metabolites influence neuronal development, mood regulation, and inflammatory pathways.	Epigenetic regulation, blood–brain barrier modulation, immune regulation, enteroendocrine signaling, and neurotransmitter synthesis	Cryan et al., 2019; Needham et al., 2020; Cheng et al., 2024; Tang et al., 2022
**Dietary modulation of microbiome–**	Dietary interventions, omega-3	Nutritional interventions modify microbial composition and can prevent or attenuate	Increased beneficial microbial taxa, SCFA production, reduced	Provensi et al., 2019; Robertson et al., 2017; Horn et

**brain communication**	supplementation, fiber/prebiotic approaches, and stress models	stress-associated cognitive and behavioral changes during adolescence.	inflammation, improved neurogenesis, and altered stress signaling	al., 2022; Bogachuk et al., 2024
**Microbiome, stress, and early-life adversity**	Early-life stress models, psychosocial stress cohorts, inflammatory profiling	Early adversity alters microbiome composition and interacts with stress systems to increase vulnerability to psychiatric outcomes.	HPA-axis activation, inflammatory signaling, immune dysregulation, altered microbial metabolites, and impaired neuroplasticity	Beurel and Nemeroff, 2024; Koss and Gunnar, 2018; Warren et al., 2024
**Microbiome regulation of reward and addiction-related pathways**	Behavioral models of reward, dopamine pathway analysis, substance-use related microbiome studies	Microbiome alterations influence reward sensitivity, dopamine signaling, and vulnerability to substance-use-related behaviors, processes particularly relevant during adolescent brain maturation.	Dopaminergic signaling, vagal communication, microbial metabolites, mesolimbic pathway regulation, and neuroimmune interactions	Hamamah et al., 2022; Barbu et al., 2025; Browning et al., 2024
**Sex differences, puberty, and microbiome–hormone interactions**	Sex-stratified microbiome analyses, hormone manipulation studies, pubertal development studies	Sex steroids shape microbial composition, while microbial communities influence hormone metabolism, contributing to sexually dimorphic	Estrobolome activity, androgen-associated microbial patterns, immune modulation, bile acid metabolism, and hormone-	Sisk and Zehr, 2005; Elderman et al., 2018; Kaliannan et al., 2018; Vemuri et al., 2019; Shobeiri et al., 2022

		neurodevelopmental outcomes.	dependent microbial signaling	
**Human adolescent microbiome studies and translational evidence**	Longitudinal cohorts, 16S rRNA sequencing, shotgun metagenomics, metabolomics, behavioral assessments	Human studies demonstrate developmental changes in microbial diversity and associations between microbiome composition, mental health, metabolism, and neurodevelopmental outcomes.	Microbial ecology, diet-dependent metabolites, immune pathways, endocrine regulation, and host–microbe interactions	Hollister et al., 2015; Laue et al., 2022; Mancabelli et al., 2024; Mohr et al., 2024
**Microbiome–immune–brain communication**	Immune profiling, microglial analysis, inflammatory pathway assessment	Microbial signals regulate immune development and microglial function, linking intestinal ecology with neurodevelopment and disease susceptibility.	Microglial maturation, cytokine signaling, intestinal permeability, immune tolerance, and neuroinflammation	Cook and Prinz, 2022; Park and Im, 2022; Morais et al., 2021
**Clinical and therapeutic approaches targeting the microbiome**	Probiotic, psychobiotic, synbiotic, and microbiota-targeted intervention studies	Modulation of microbial communities represents a potential strategy to influence stress, anxiety, depression, and cognitive outcomes, although adolescent-specific trials remain limited.	Restoration of microbial diversity, SCFA production, serotonin pathways, immune regulation, and gut barrier function	Heidarzadeh-Rad et al., 2020; Merkouris et al., 2024; Kumar et al., 2023
